# Supplementary material for: Computer simulation of Cerebral Arteriovenous Malformation—validation analysis of hemodynamics parameters
Source: PeerJ. 2017 Jan 26;5:e2724. doi: 10.7717/peerj.2724 (PMC5274518; doi:10.7717/peerj.2724)
Supplement: Supplemental Information 1 [file peerj-05-2724-s003.pdf]

XLSTAT 2014.4.06 - ANCOVA - on 8/11/2014 at 10:52:59 AM
Y / Quantitative: Workbook = statisticasxlsx.xlsx / Sheet = inputdata / Range = inputdata!$B$5:$B$32
X / Quantitative: Workbook = statisticasxlsx.xlsx / Sheet = inputdata / Range = inputdata!$C$5:$C$32
X / Qualitative: Workbook = statisticasxlsx.xlsx / Sheet = inputdata / Range = inputdata!$B$5:$B$32 /
Constraints: an=0
Confidence interval (%): 95
Tolerance: 0.0001
Use least squares means: Yes

Summary statistics:

Variable	)bservation	with missin	thout missi	Minimum	Maximum	Mean	td. deviation
0.03	27	0	27	0.010	34.000	1.430	6.511
0.03	27	0	27	0.000	0.382	0.175	0.140

Variable	Categories	requencie	%
0.03	0.01	2	7.407
	0.014	1	3.704
	0.015	2	7.407
	0.02	1	3.704
	0.03	2	7.407
	0.035	1	3.704
	0.05	2	7.407
	0.15	1	3.704
	0.19	1	3.704
	0.24	1	3.704
	0.267	1	3.704
	0.28	2	7.407
	0.281	1	3.704
	0.289	1	3.704
	0.301	1	3.704
	0.31	1	3.704
	0.32	2	7.407
	0.35	1	3.704
	0.36	1	3.704
	0.4	1	3.704
	34	1	3.704

Correlation matrix:

Variables	0.03	0.03-0.01	0.03-0.014	0.03-0.015	0.03-0.02	0.03-0.03	0.03-0.035	0.03-0.05
0.03	**1.000**	-0.358	-0.235	-0.339	-0.224	-0.308	-0.207	-0.257

0.03-0.01	-0.358	**1.000**	-0.055	-0.080	-0.055	-0.080	-0.055	-0.080
0.03-0.014	-0.235	-0.055	**1.000**	-0.055	-0.038	-0.055	-0.038	-0.055
0.03-0.015	-0.339	-0.080	-0.055	**1.000**	-0.055	-0.080	-0.055	-0.080
0.03-0.02	-0.224	-0.055	-0.038	-0.055	**1.000**	-0.055	-0.038	-0.055
0.03-0.03	-0.308	-0.080	-0.055	-0.080	-0.055	**1.000**	-0.055	-0.080
0.03-0.035	-0.207	-0.055	-0.038	-0.055	-0.038	-0.055	**1.000**	-0.055
0.03-0.05	-0.257	-0.080	-0.055	-0.080	-0.055	-0.080	-0.055	**1.000**
0.03-0.15	-0.079	-0.055	-0.038	-0.055	-0.038	-0.055	-0.038	-0.055
0.03-0.19	0.004	-0.055	-0.038	-0.055	-0.038	-0.055	-0.038	-0.055
0.03-0.24	0.075	-0.055	-0.038	-0.055	-0.038	-0.055	-0.038	-0.055
0.03-0.267	0.114	-0.055	-0.038	-0.055	-0.038	-0.055	-0.038	-0.055
0.03-0.28	0.187	-0.080	-0.055	-0.080	-0.055	-0.080	-0.055	-0.080
0.03-0.281	0.131	-0.055	-0.038	-0.055	-0.038	-0.055	-0.038	-0.055
0.03-0.289	0.145	-0.055	-0.038	-0.055	-0.038	-0.055	-0.038	-0.055
0.03-0.301	0.165	-0.055	-0.038	-0.055	-0.038	-0.055	-0.038	-0.055
0.03-0.31	0.165	-0.055	-0.038	-0.055	-0.038	-0.055	-0.038	-0.055
0.03-0.32	0.297	-0.080	-0.055	-0.080	-0.055	-0.080	-0.055	-0.080
0.03-0.35	0.249	-0.055	-0.038	-0.055	-0.038	-0.055	-0.038	-0.055
0.03-0.36	0.263	-0.055	-0.038	-0.055	-0.038	-0.055	-0.038	-0.055
0.03-0.4	0.294	-0.055	-0.038	-0.055	-0.038	-0.055	-0.038	-0.055
0.03-34	0.219	-0.055	-0.038	-0.055	-0.038	-0.055	-0.038	-0.055
0.03	0.240	-0.063	-0.043	-0.063	-0.043	-0.062	-0.043	-0.061

Multicolinearity statistics:

Statistic	0.03	0.03-0.01	0.03-0.014	0.03-0.015	0.03-0.02	0.03-0.03	0.03-0.035	0.03-0.05
Tolerance	0.000	0.872	0.945	0.885	0.950	0.905	0.957	0.934
VIF	9129.876	1.147	1.059	1.130	1.053	1.105	1.045	1.071

Regression of variable 0.03:

Goodness of fit statistics:

Observatio	27.000
Sum of we	27.000
DF	5.000
R^2	1.000
Adjusted R	1.000
MSE	0.000
RMSE	0.000
MAPE	0.000
DW	
Cp	22.000
AIC	

SBC	
PC	0.000

Analysis of variance:

Source	DF	ım of squar	lean square	F	Pr > F
Model	21	1102.087	52.480		
Error	5	0.000	0.000		
Corrected	26	1102.087			

Computed against model Y=Mean(Y)

Model parameters:

Source	Value	:andard err	t	Pr > |t|	er bound (9	er bound (95%)
Intercept	34.000	0.000				
0.03	0.000	0.000				
0.03-0.01	-33.990	0.000				
0.03-0.014	-33.986	0.000				
0.03-0.015	-33.985	0.000				
0.03-0.02	-33.980	0.000				
0.03-0.03	-33.970	0.000				
0.03-0.035	-33.965	0.000				
0.03-0.05	-33.950	0.000				
0.03-0.15	-33.850	0.000				
0.03-0.19	-33.810	0.000				
0.03-0.24	-33.760	0.000				
0.03-0.267	-33.733	0.000				
0.03-0.28	-33.720	0.000				
0.03-0.281	-33.719	0.000				
0.03-0.289	-33.711	0.000				
0.03-0.301	-33.699	0.000				
0.03-0.31	-33.690	0.000				
0.03-0.32	-33.680	0.000				
0.03-0.35	-33.650	0.000				
0.03-0.36	-33.640	0.000				
0.03-0.4	-33.600	0.000				
0.03-34	0.000	0.000				

Equation of the model:

0.03 = 34.0000000002655-8.06353889602984E-10*0.03-33.9900000002646*0.03-0.01-33.9860000(

Standardized coefficients:

Source	Value	:andard erro	t	Pr > |t|	er bound (9	er bound (95%)
0.03	0.000	0.000				
0.03-0.01	-1.393	0.000				
0.03-0.014	-1.005	0.000				
0.03-0.015	-1.393	0.000				
0.03-0.02	-1.004	0.000				
0.03-0.03	-1.392	0.000				
0.03-0.035	-1.004	0.000				
0.03-0.05	-1.392	0.000				
0.03-0.15	-1.001	0.000				
0.03-0.19	-0.999	0.000				
0.03-0.24	-0.998	0.000				
0.03-0.267	-0.997	0.000				
0.03-0.28	-1.382	0.000				
0.03-0.281	-0.997	0.000				
0.03-0.289	-0.996	0.000				
0.03-0.301	-0.996	0.000				
0.03-0.31	-0.996	0.000				
0.03-0.32	-1.381	0.000				
0.03-0.35	-0.995	0.000				
0.03-0.36	-0.994	0.000				
0.03-0.4	-0.993	0.000				
0.03-34	0.000	0.000				

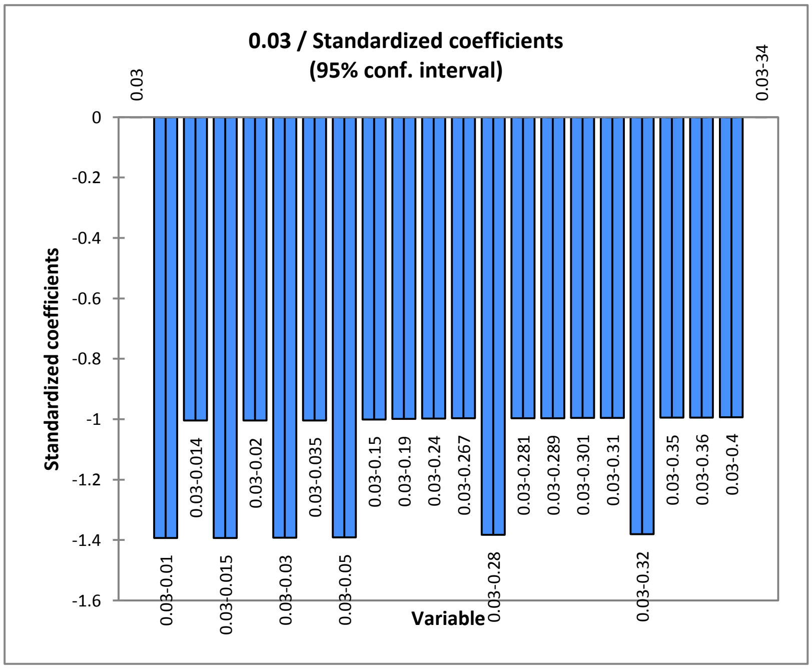

Predictions and residuals:

Observatior	Weight	0.03	0.03	Pred(0.03)	Residual	Std. residua	entized resiv. on pred.
Obs1	1	0.010	0.015	0.015	0.000	0.000	1.000
Obs2	1	0.002	0.010	0.010	0.000	0.000	1.000
Obs3	1	0.120	0.150	0.150	0.000	0.000	0.000
Obs4	1	0.050	0.050	0.050	0.000	0.000	1.000
Obs5	1	0.020	0.030	0.030	0.000	0.000	1.000
Obs6	1	0.010	0.014	0.014	0.000	0.000	0.000
Obs7	1	0.320	0.320	0.320	0.000	0.000	1.000
Obs8	1	0.291	0.301	0.301	0.000	0.000	0.000
Obs9	1	0.267	0.281	0.281	0.000	0.000	0.000
Obs10	1	0.360	0.360	0.360	0.000	0.000	0.000
Obs11	1	0.277	0.289	0.289	0.000	0.000	0.000
Obs12	1	0.255	0.267	0.267	0.000	0.000	0.000
Obs13	1	0.320	0.320	0.320	0.000	0.000	1.000
Obs14	1	0.265	0.280	0.280	0.000	0.000	1.000
Obs15	1	0.228	0.240	0.240	0.000	0.000	0.000
Obs16	1	0.382	0.400	0.400	0.000	0.000	0.000
Obs17	1	0.329	34.000	34.000	0.000	0.000	0.000
Obs18	1	0.291	0.310	0.310	0.000	0.000	0.000
Obs19	1	0.350	0.350	0.350	0.000	0.000	0.000
Obs20	1	0.268	0.280	0.280	0.000	0.000	1.000
Obs21	1	0.178	0.190	0.190	0.000	0.000	0.000
Obs22	1	0.050	0.050	0.050	0.000	0.000	1.000
Obs23	1	0.030	0.035	0.035	0.000	0.000	0.000
Obs24	1	0.018	0.020	0.020	0.000	0.000	0.000
Obs25	1	0.030	0.030	0.030	0.000	0.000	1.000
Obs26	1	0.010	0.015	0.015	0.000	0.000	1.000
Obs27	1	0.000	0.010	0.010	0.000	0.000	1.000

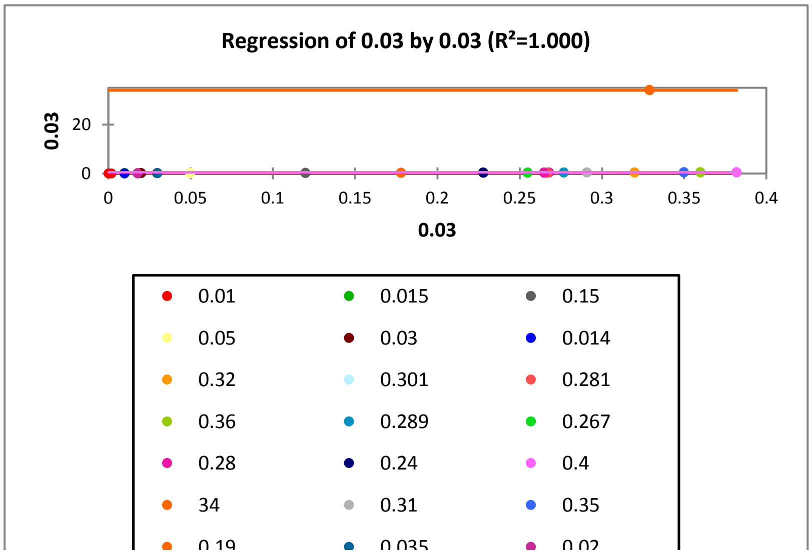

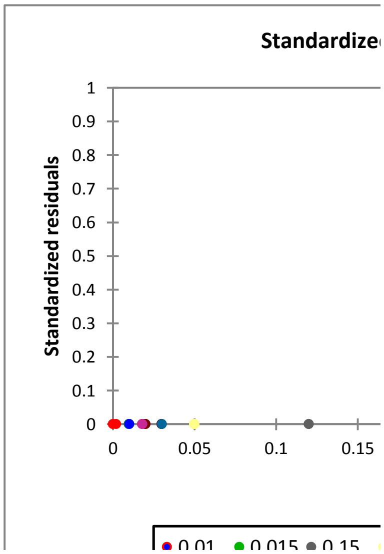

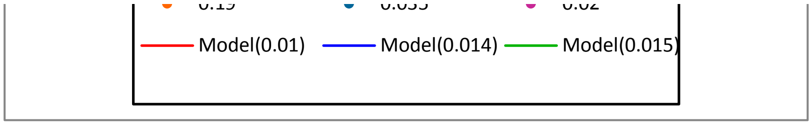

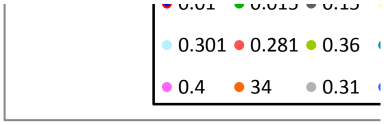

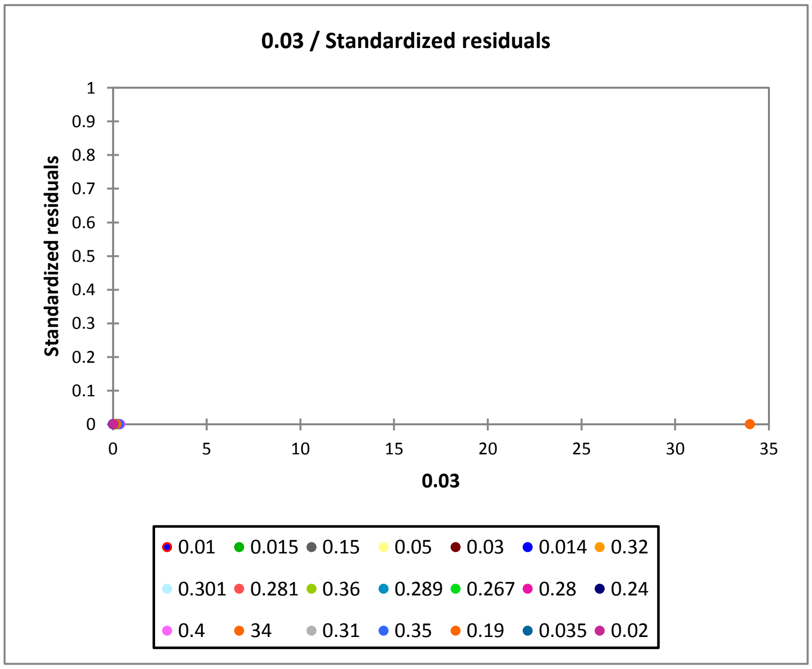

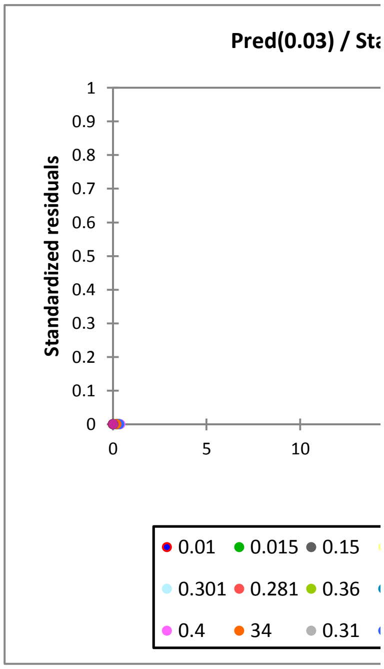

Means charts:

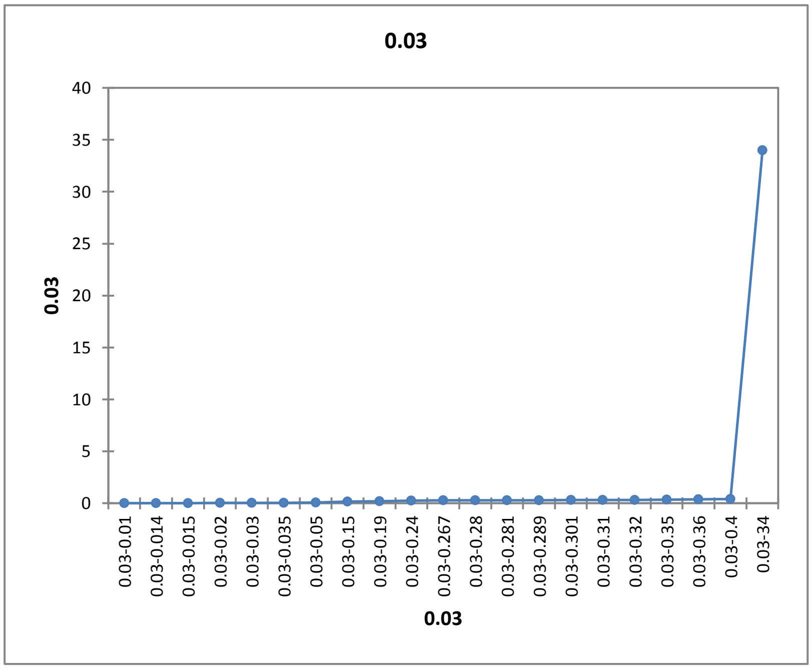

2 / 27 rows and 1 column
2 / 27 rows and 1 column
/ 27 rows and 1 column

0.03-0.15	0.03-0.19	0.03-0.24	0.03-0.267	0.03-0.28	0.03-0.281	0.03-0.289	0.03-0.301	0.03-0.31	0.03-0.32
-0.079	0.004	0.075	0.114	0.187	0.131	0.145	0.165	0.165	0.297

-0.055	-0.055	-0.055	-0.055	-0.080	-0.055	-0.055	-0.055	-0.055	-0.080
-0.038	-0.038	-0.038	-0.038	-0.055	-0.038	-0.038	-0.038	-0.038	-0.055
-0.055	-0.055	-0.055	-0.055	-0.080	-0.055	-0.055	-0.055	-0.055	-0.080
-0.038	-0.038	-0.038	-0.038	-0.055	-0.038	-0.038	-0.038	-0.038	-0.055
-0.055	-0.055	-0.055	-0.055	-0.080	-0.055	-0.055	-0.055	-0.055	-0.080
-0.038	-0.038	-0.038	-0.038	-0.055	-0.038	-0.038	-0.038	-0.038	-0.055
-0.055	-0.055	-0.055	-0.055	-0.080	-0.055	-0.055	-0.055	-0.055	-0.080
**1.000**	-0.038	-0.038	-0.038	-0.055	-0.038	-0.038	-0.038	-0.038	-0.055
-0.038	**1.000**	-0.038	-0.038	-0.055	-0.038	-0.038	-0.038	-0.038	-0.055
-0.038	-0.038	**1.000**	-0.038	-0.055	-0.038	-0.038	-0.038	-0.038	-0.055
-0.038	-0.038	-0.038	**1.000**	-0.055	-0.038	-0.038	-0.038	-0.038	-0.055
-0.055	-0.055	-0.055	-0.055	**1.000**	-0.055	-0.055	-0.055	-0.055	-0.080
-0.038	-0.038	-0.038	-0.038	-0.055	**1.000**	-0.038	-0.038	-0.038	-0.055
-0.038	-0.038	-0.038	-0.038	-0.055	-0.038	**1.000**	-0.038	-0.038	-0.055
-0.038	-0.038	-0.038	-0.038	-0.055	-0.038	-0.038	**1.000**	-0.038	-0.055
-0.038	-0.038	-0.038	-0.038	-0.055	-0.038	-0.038	-0.038	**1.000**	-0.055
-0.055	-0.055	-0.055	-0.055	-0.080	-0.055	-0.055	-0.055	-0.055	**1.000**
-0.038	-0.038	-0.038	-0.038	-0.055	-0.038	-0.038	-0.038	-0.038	-0.055
-0.038	-0.038	-0.038	-0.038	-0.055	-0.038	-0.038	-0.038	-0.038	-0.055
-0.038	-0.038	-0.038	-0.038	-0.055	-0.038	-0.038	-0.038	-0.038	-0.055
-0.038	-0.038	-0.038	-0.038	-0.055	-0.038	-0.038	-0.038	-0.038	-0.055
-0.039	-0.038	-0.037	-0.036	-0.051	-0.035	-0.035	-0.035	-0.034	-0.049

0.03-0.15	0.03-0.19	0.03-0.24	0.03-0.267	0.03-0.28	0.03-0.281	0.03-0.289	0.03-0.301	0.03-0.31	0.03-0.32
0.994	1.000	0.994	0.987	0.965	0.983	0.979	0.973	0.973	0.912
1.006	1.000	1.006	1.013	1.036	1.017	1.021	1.028	1.028	1.097

002574*0.03-0.014-33.9850000002574*0.03-0.015-33.980000000251*0.03-0.02-33.9700000002454*0.03-0.03

ound 95%	ound 95%	n pred. (Ol	nd 95% (Ol	nd 95% (Observation)

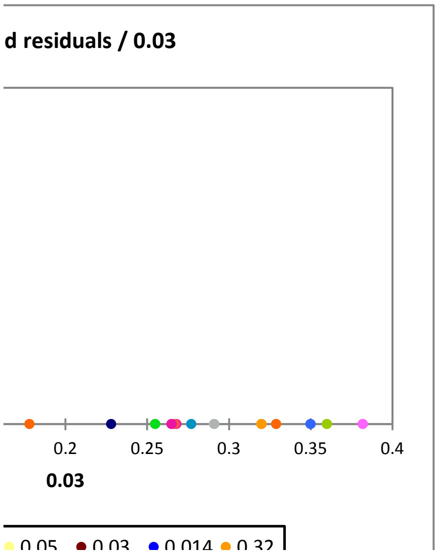

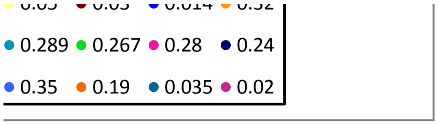
0.289 0.267 0.28 0.24
0.35 0.19 0.035 0.02

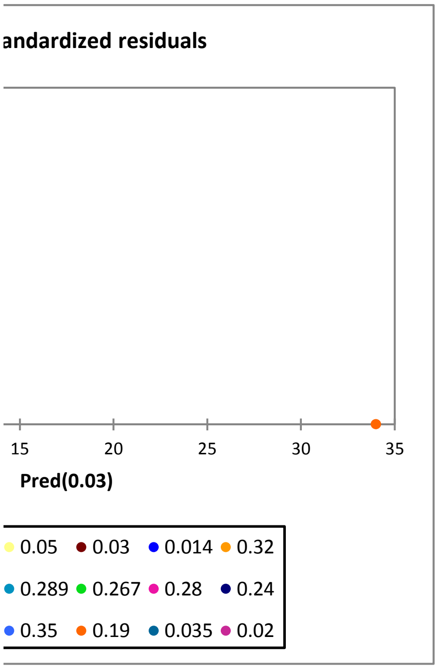
andardized residuals
15 20 25 30 35
Pred(0.03)
0.05 0.03 0.014 0.32
0.289 0.267 0.28 0.24
0.35 0.19 0.035 0.02

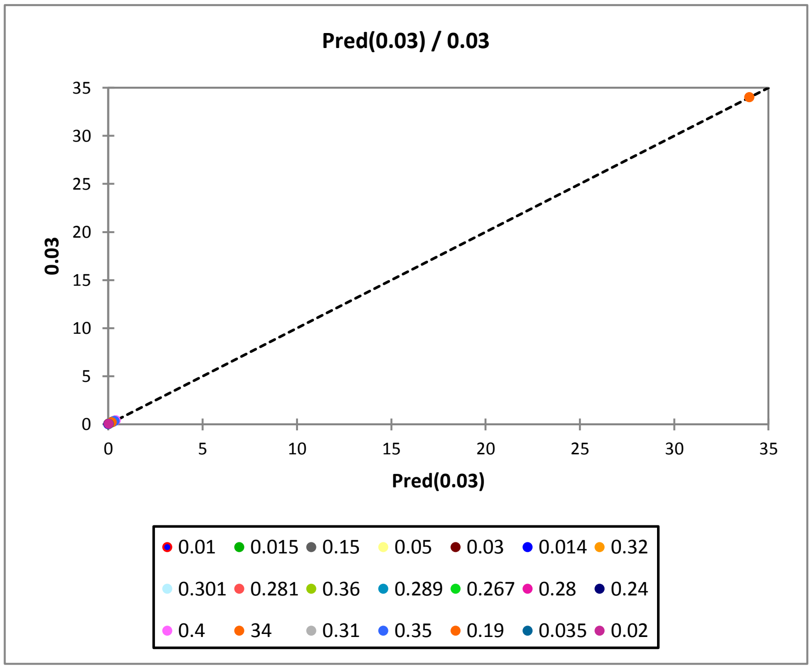
Pred(0.03) / 0.03
0.03
0 5 10 15 20 25 30 35
Pred(0.03)
0.01 0.015 0.15 0.05 0.03 0.014 0.32
0.301 0.281 0.36 0.289 0.267 0.28 0.24
0.4 34 0.31 0.35 0.19 0.035 0.02

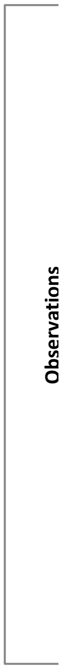
Observations

0.03-0.35	0.03-0.36	0.03-0.4	0.03-34	0.03
0.249	0.263	0.294	0.219	0.240

-0.055	-0.055	-0.055	-0.055	-0.063
-0.038	-0.038	-0.038	-0.038	-0.043
-0.055	-0.055	-0.055	-0.055	-0.063
-0.038	-0.038	-0.038	-0.038	-0.043
-0.055	-0.055	-0.055	-0.055	-0.062
-0.038	-0.038	-0.038	-0.038	-0.043
-0.055	-0.055	-0.055	-0.055	-0.061
-0.038	-0.038	-0.038	-0.038	-0.039
-0.038	-0.038	-0.038	-0.038	-0.038
-0.038	-0.038	-0.038	-0.038	-0.037
-0.038	-0.038	-0.038	-0.038	-0.036
-0.055	-0.055	-0.055	-0.055	-0.051
-0.038	-0.038	-0.038	-0.038	-0.035
-0.038	-0.038	-0.038	-0.038	-0.035
-0.038	-0.038	-0.038	-0.038	-0.035
-0.038	-0.038	-0.038	-0.038	-0.034
-0.055	-0.055	-0.055	-0.055	-0.049
**1.000**	-0.038	-0.038	-0.038	-0.033
-0.038	**1.000**	-0.038	-0.038	-0.033
-0.038	-0.038	**1.000**	-0.038	-0.032
-0.038	-0.038	-0.038	**1.000**	1.000
-0.033	-0.033	-0.032	1.000	**1.000**

0.03-0.35	0.03-0.36	0.03-0.4	0.03-34
0.938	0.931	0.913	0.952
1.066	1.074	1.095	1.050

[illegible]-33.9650000002413*0.03-0.035-33.9500000002252*0.03-0.05-33.8500000001687*0.03-0.15-33.81000000012

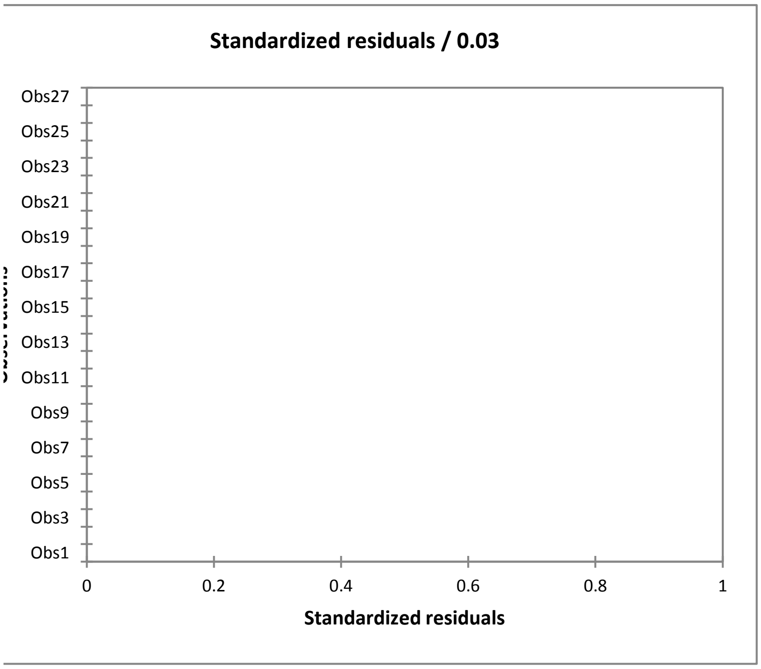
Standardized residuals / 0.03
Obs27
Obs25
Obs23
Obs21
Obs19
Obs17
Obs15
Obs13
Obs11
Obs9
Obs7
Obs5
Obs3
Obs1
0
0.2
0.4
0.6
0.8
1
Standardized residuals

.19*0.03-0.19-33.7600000000816*0.03-0.24-33.7330000000598*0.03-0.267-33.7200000000506*0.03-0.28-33.[illegible]

7190000000502*0.03-0.281-33.7110000000421*0.03-0.289-33.6990000000308*0.03-0.301-33.6900000000308

3*0.03-0.31-33.6800000000075*0.03-0.32-33.6499999999832*0.03-0.35-33.6399999999752*0.03-0.36-33.599

9999999574*0.03-0.4
